# Adapted Protocol for *Saccharibacteria* Cocultivation: Two New Members Join the Club of Candidate Phyla Radiation

**DOI:** 10.1128/spectrum.01069-21

**Published:** 2021-12-22

**Authors:** Ahmad Ibrahim, Mohamad Maatouk, Andriamiharimamy Rajaonison, Rita Zgheib, Gabriel Haddad, Jacques Bou Khalil, Didier Raoult, Fadi Bittar

**Affiliations:** a IHU Méditerranée Infection, Marseille, France; b Aix-Marseille Université, IRD, APHM, MEPHI, Marseille, France; c Aix-Marseille Université, IRD, APHM, SSA, VITROME, Marseille, France; Temasek Life Sciences Laboratory

**Keywords:** candidate phyla radiation, *Saccharibacteria*, *Schaalia odontolytica*, coculture, Minimicrobia, protocol, real-time PCR

## Abstract

The growing application of metagenomics to different ecological and microbiome niches in recent years has enhanced our knowledge of global microbial biodiversity. Among these abundant and widespread microbes, the candidate phyla radiation (CPR) group has been recognized as representing a large proportion of the microbial kingdom (>26%). CPR are characterized by their obligate symbiotic or exoparasitic activity with other microbial hosts, mainly bacteria. Currently, isolating CPR is still considered challenging for microbiologists. The idea of this study was to develop an adapted protocol for the coculture of CPR with a suitable bacterial host. Based on various sputum samples, we tried to enrich CPR (*Saccharibacteria* members) and to cocultivate them with pure hosts (Schaalia odontolytica). This protocol was monitored by TaqMan real-time quantitative PCR (qPCR) using a system specific for *Saccharibacteria* designed in this study, as well as by electron microscopy and sequencing. We succeeded in coculturing and sequencing the complete genomes of two new *Saccharibacteria* species, “*Candidatus* Minimicrobia naudis” and “*Candidatus* Minimicrobia vallesae.” In addition, we noticed a decrease in the *C_T_* values of *Saccharibacteria* and a significant multiplication through their physical association with Schaalia odontolytica strains in the enriched medium that we developed. This work may help bridge gaps in the genomic database by providing new CPR members, and in the future, their currently unknown characteristics may be revealed.

**IMPORTANCE** In this study, the first TaqMan real-time quantitative PCR (qPCR) system, targeting *Saccharibacteria* phylum, has been developed. This technique can specifically quantify *Saccharibacteria* members in any sample of interest in order to investigate their prevalence. In addition, another easy, specific, and sensitive protocol has been developed to maintain the viability of *Saccharibacteria* cells in an enriched medium with their bacterial host. The use of this protocol facilitates subsequent studies of the phenotypic characteristics of CPR and their physical interactions with bacterial species, as well as the sequencing of new genomes to improve the current database.

## INTRODUCTION

Over the past 2 decades, the fast progress of molecular methods and the intensive use of both total and targeted metagenomics (mainly using the 16S rRNA gene) have led to the recognition of new microorganisms which were not previously reported (1, 2). These recently described microbes, which now represent a huge and diverse proportion of the microbial domain, are generally microorganisms that have not yet been cultured (1). Following each major discovery, and according to a recent classification based on whole-genome content analyses, the CPR is beginning to appear as a new division in the rhizome of life, independent from classical bacteria (3, 4). Since these microbes are not present in a pure cultivable state, their phenotypic characteristics remain incompletely defined (5). All known data are simply extracted from predictions based on bioinformatics analyses, which encourages microbiologists to culture them (1, 5). However, many difficulties limit their culture, such as slow growth/division, the need for specific metabolites in the final medium, and growth inhibition by other dominant microorganisms or, inversely, the need for an obligatory association with another microorganism serving as a host in order to flourish (1, 2, 6, 7).

Recent studies on microbial diversity in human and environmental samples based on whole-metagenomics analyses have made it possible to identify a new group of microorganisms that are not well recognized by using the 16S rRNA gene. These microbes, called the CPR, continue to be resistant to culture (8). This group is comprised of more than 73 new phyla and represents a huge proportion (more than 26%) of the bacterial domain (2, 9, 10). Although CPR members present high interindividual heterogeneity in genomic sequences, they do have certain common characteristics: they are morphologically small (100 to 300 nm) and have reduced genome sizes (usually less than 1 Mbp) (1), high percentages of hypothetical proteins (11), and a single copy of the 16S rRNA gene (8). Furthermore, CPR members have a developed cell membrane close to that of Gram-positive bacteria (11), as well as limited and unknown/undetailed biosynthetic and metabolic capacities (12). In addition, they are enriched in proteins involved in cell-cell interactions, such as the presence of pili belonging to the type IV secretion system (13). These proteins allow CPR members to be attached to their respective hosts, characterizing their lifestyle, which appears to be either an exosymbiotic or an exoparasitic relationship (6, 7, 13).

Recently, it has been suggested that CPR coevolved with bacteria (and not from bacteria), based on the distribution and diversity of their protein families (4, 11). Recent studies have shown that CPR are unable to synthesize nucleotides *de novo* and that they retain only the genes essential for their survival (11, 14). In fact, CPR seem to behave in a different, particular way (a nontraditional biological process), with their own ribosomal structures, and introns are present in their tRNA and 16S rRNA sequences (12). Analysis of the genomes available in the NCBI (National Centre for Biotechnology Information) database has led to the prediction of certain phenotypic characteristics unique to this group of microbes. These characteristics include antibiotic resistance (15), their natural resistance to bacteriophage despite the absence of the CRISPR viral defense in their genomes, which is due to the lack of viral receptors in their cell membrane (16), and the presence of different proteins involved in quorum-sensing phenomena and cell-cell communication (17). None of these characteristics, however, have yet been confirmed *in vitro.*

*Saccharibacteria*, or TM7, is the most-studied CPR superphylum. It was first described through metagenomics study of neglected uncultured bacteria from multiple metagenomes (18) and was named due to its metabolism of sugar (18, 19). Sequences belonging to this superphylum have been systematically detected in various environmental and ecological samples, including samples from soil, freshwater lakes, dolphin teeth, termite guts, activated sludge, etc. (20–22). In addition, metagenomics studies have shown that members of TM7 are also present in the human microbiome, including the intestinal, oral, urinary, cutaneous, blood, and vaginal microbiota (11, 19, 23–26). Various studies have shown that *Saccharibacteria* members are associated with various human mucosa-related diseases, such as vaginosis, periodontitis, and bowel disease (6, 23, 27).

To date, a few members of *Saccharibacteria* have been cocultured with different bacterial hosts, most often Schaalia odontolytica, *Actinomyces* spp., Cellulosimicrobium cellulans, and Arachnia propionica (1, 2, 6, 28). The first cocultured TM7 strain was reported in 2014 by Soro et al., without genomic information (29). In addition, based on streptomycin resistance selection, TM7x HMT-952 (TM7x hereinafter; also known as “*Candidatus* Nanosynbacter lyticus”) was among the first TM7 strains to be cultivated and sequenced with its bacterial host, in 2015 (6).

In order to expand our knowledge about this superphylum and to improve its phenotypic characterization, culture is essential. The aims of this study were to develop a specific TaqMan real-time quantitative PCR (qPCR) for quantifying *Saccharibacteria* spp., to improve the enrichment broth, and then to develop an easy and reproducible protocol for *Saccharibacteria* cocultivation. This was based on enriching the strains belonging to the *Saccharibacteria* species recovered from a human oral sample and then cocultivating them with a mixture of strains of a bacterium of interest (here, Schaalia odontolytica) to maintain their viability.

## RESULTS

### Specificity of the real-time PCR system.

In order to quantify *Saccharibacteria* spp., we have managed to design a specific TaqMan real-time qPCR system. The specificity of our designated qPCR system was confirmed using a collection of DNAs from bacterial/fungal species and human samples (see Materials and Methods). All bacterial and fungal DNA samples, as well as the 25 stool samples used, were negative by our TaqMan qPCR system and by primers 580-F/1177-R, which are specific for *Saccharibacteria*. For greater accuracy, we tested 25 different sputum samples in duplicates. All samples were positive by standard PCR and by our designated real-time PCR, with cycle threshold (*C_T_*) values ranging between 17.02 (±0.2 [mean ± standard deviation]) and 23.57 (±0.2). In addition, the BLASTn analysis of the amplicons sequenced by Sanger sequencing shows that they all matched with different *Saccharibacteria* 23S rRNA genes. This system can amplify 126-bp fragments of the 23S rRNA gene, which serves as a specific marker for all *Saccharibacteria* spp. Moreover, this specificity was reconfirmed by selecting all additional complete genomes available on the NCBI database between 1 December 2020 and 1 June 2021. This system was able to amplify 34/35 tested genomes (the same conserved zone for all genomes).

### Isolation and coculture of *Saccharibacteria* species and quantification test.

**FIG 1:**
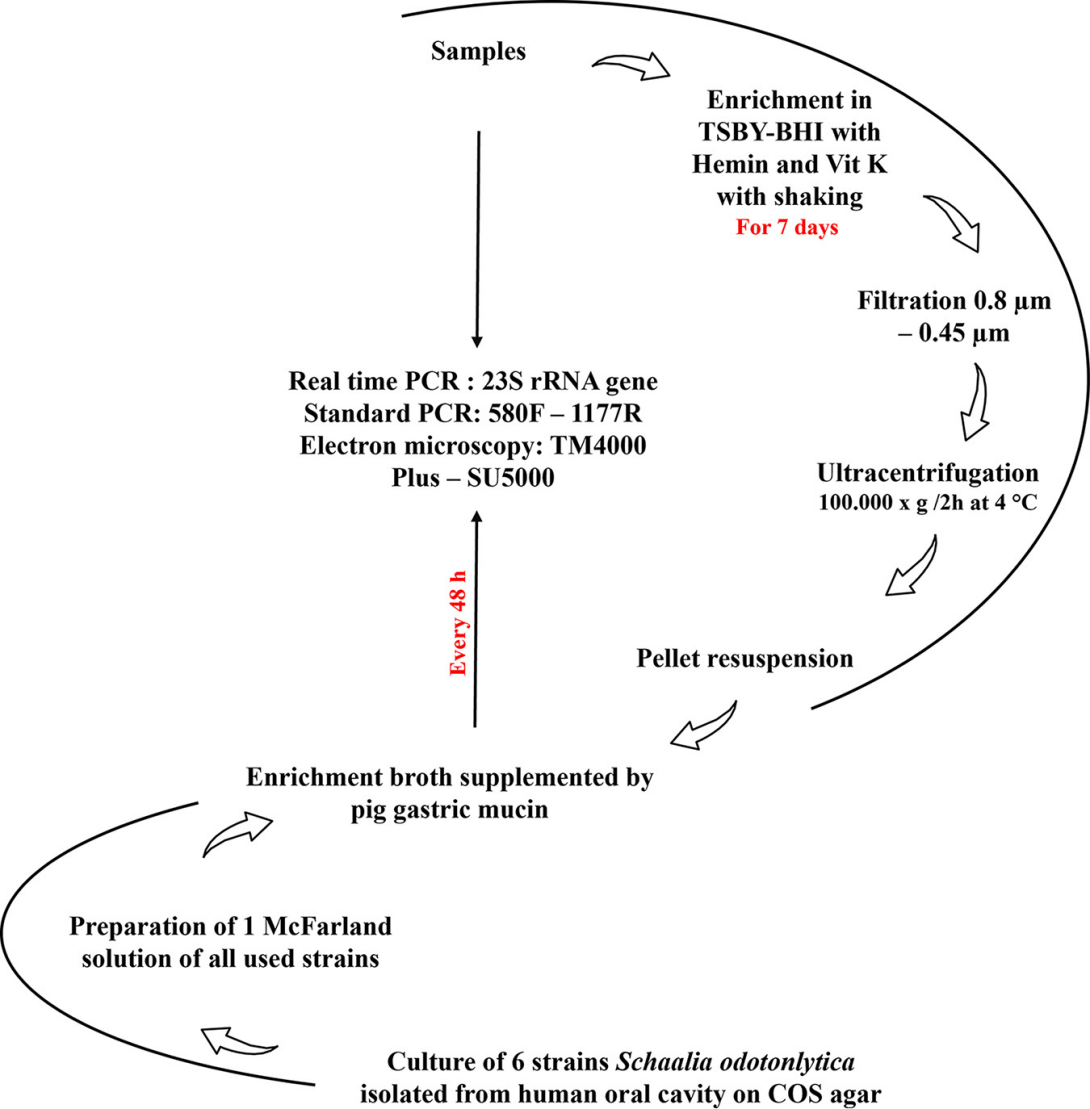
Summary of the *Saccharibacteria* coculture protocol used in this study.

**FIG 2:**
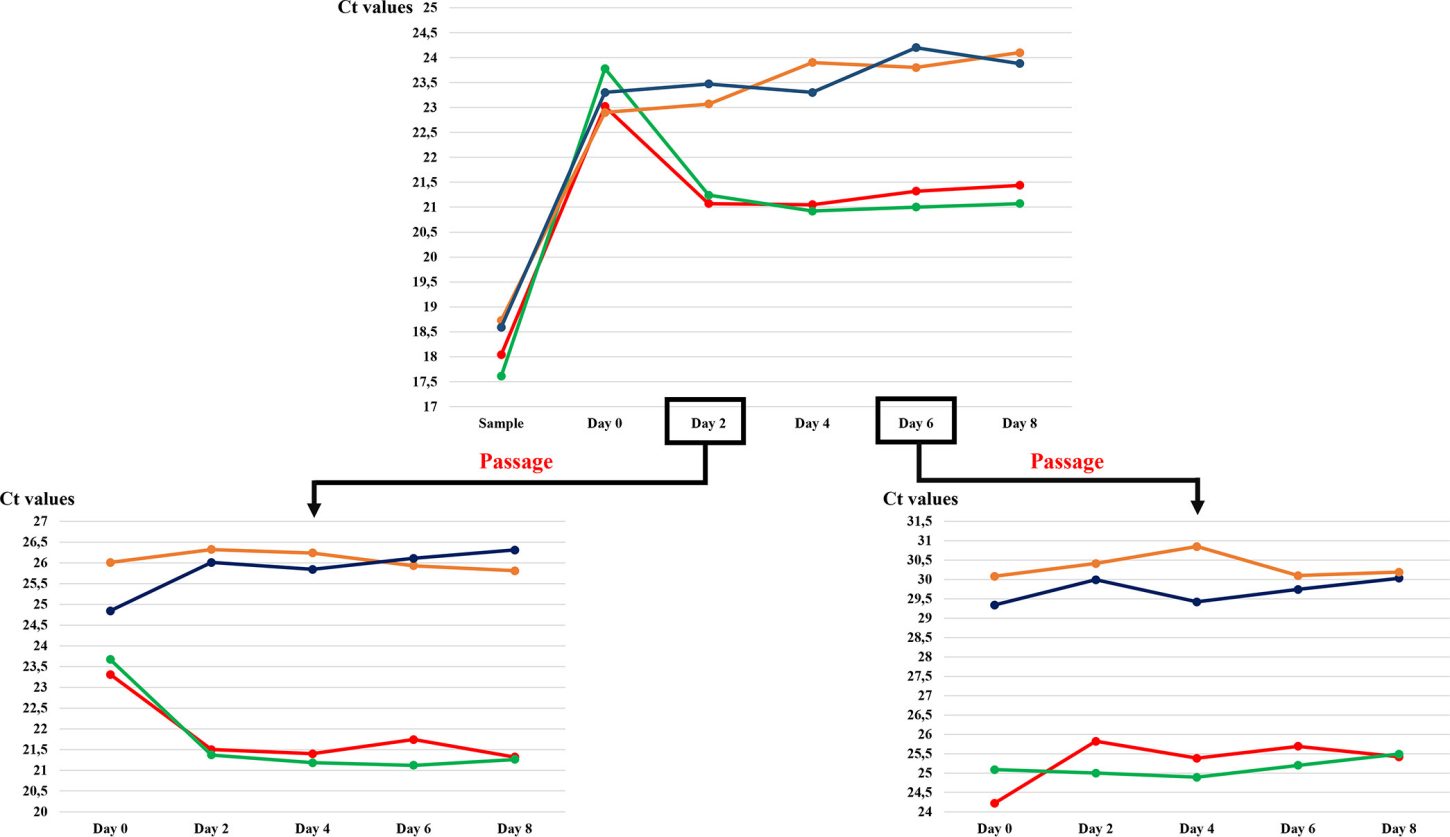
Graphic representation showing (top) the *C_T_* variations of *Saccharibacteria* between each coculture condition tested in this study, (bottom left) the *C_T_* variations of each coculture condition after first passage at day 2, and (bottom right) the *C_T_* variations of each coculture condition after first passage at day 6. The coculture of the first sputum sample with Schaalia odontolytica is represented in red, and the second one is represented in green. For the cocultures with *Streptomyces* strains, the anaerobic conditions are indicated in blue, and the aerobic conditions in orange.

**FIG 3:**
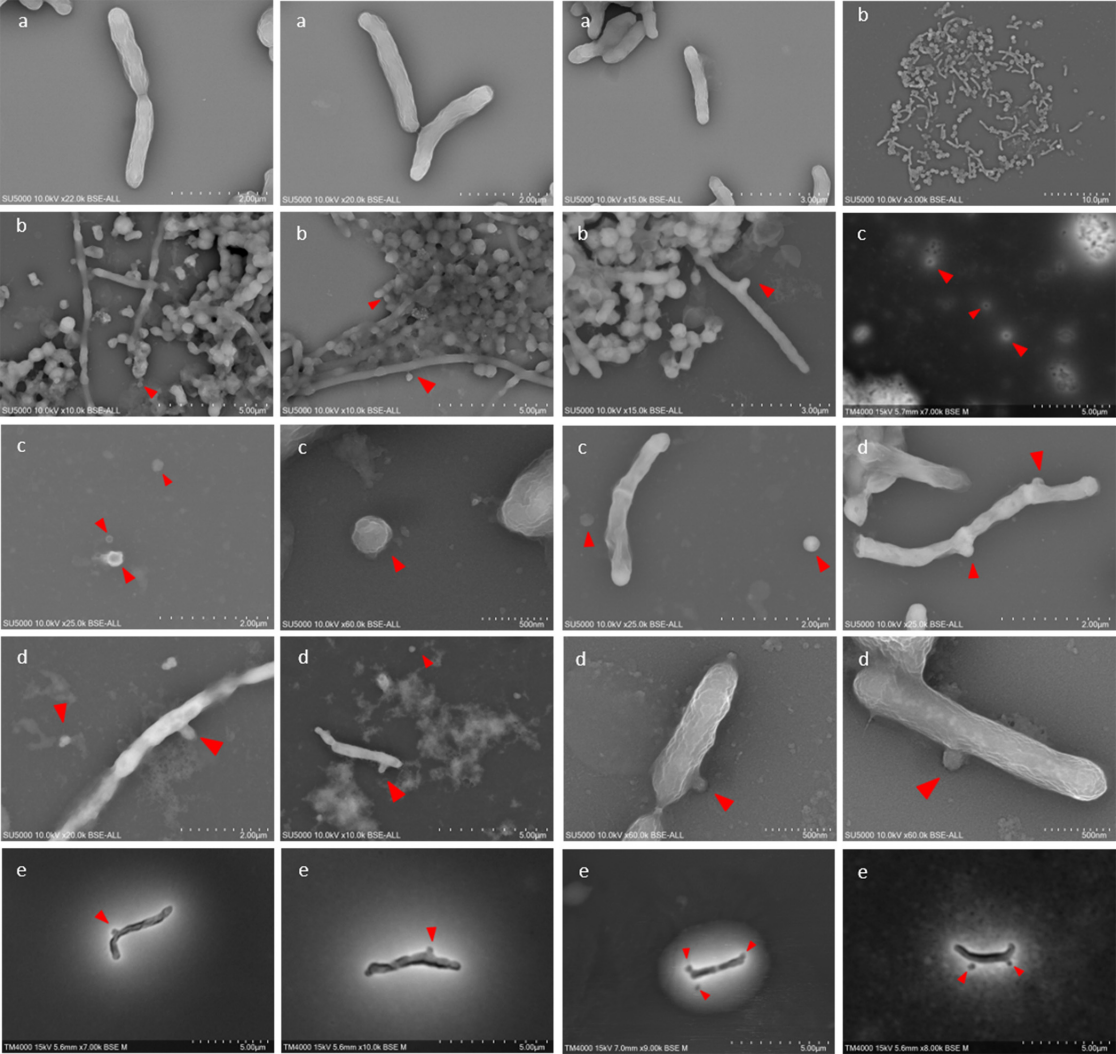
Electron microscopy micrographs showing the different steps of *Saccharibacteria* coculture. (a) *Schaalia odontolytica*, the bacterial host used in this study. (b) The presence of *Saccharibacteria* in the tested samples. (c) The *Saccharibacteria* cells detached from their bacterial host after filtration. (d and e) The physical association between filtered *Saccharibacteria* cells and their new host (Schaalia odontolytica) using the Hitachi SU5000 (d) and the Hitachi TM4000Plus (e) electron microscopes. Red arrows indicate CPR cells.

Two sputum samples were used in this study. Each culture condition was quantified by our system in duplicate. After checking that these two samples were positive for *Saccharibacteria* by our specific real-time PCR (similar *C_T_* values were obtained for the two original samples tested (18.04 and 17.61, respectively), a 7-day period of enrichment in tryptic soy broth with yeast extract plus brain heart infusion (TSBY-BHI) supplemented with hemin and vitamin K was initiated. Given that CPR members have a physically reduced corpuscle, they can pass through a 0.45- to 0.22-μm filter (2), allowing efficient isolation of CPR cells for coculturing and sequencing. This was confirmed by electron microscopy (see “*Saccharibacteria* cell imaging by electron microscopy” below). In addition, after filtration, we managed to concentrate *Saccharibacteria* cells in high quantities by ultracentrifugation (Fig. 1) (2, 6). Most of the reads obtained (≈84%) by MiSeq and GridION sequencing corresponded to *Saccharibacteria* sequences. After mixing the pellet with a mixture of 6 S. odontolytica strains as bacterial hosts (1, 2, 6) and due to the protocol steps, the *C_T_* value of each sample was, respectively, 23.02 and 23.78). Coculturing was then monitored by qPCR. In both samples, we noticed a significant decrease in the *C_T_* value after 48 h of culture (21.07 and 21.24, respectively) (Fig. 2). This decrease of the *C_T_* values indicates cellular multiplication and maintenance of viability of the enriched/filtered *Saccharibacteria* spp. However, after this step and until the 8th day of culture, no significant variations in *C_T_* values were observed. The *C_T_* values remained almost stable. The presence of *Saccharibacteria* cells at each step was also confirmed by using electron microscopy (Hitachi TM4000Plus and SU5000 microscopes) to follow the presence of exosymbiotic cocci attached to several bacterial forms (Fig. 3).

To ensure that the nutrients were continuously renewed, passages were performed on the 2nd and 6th days of culture; 200 μl of the enrichment broth (containing *Saccharibacteria* cells and S. odontolytica strains) was mixed with 2.4 ml of the initial medium supplemented with pig gastric mucin (2) and incubated at 37°C under anaerobic conditions. Due to the dilution factor (200 μl in 2.4 ml), the *C_T_* values were higher on day zero of the passage (day 2 of the initial coculture) in both samples (25.07 and 24.87, respectively) (Fig. 2). We obtained comparable results: after only 48 h of incubation, the *C_T_* values were also lower (23.61 for the first sample and 23.9 for the second) than those obtained at day 0 of the first passage (Fig. 2). Conversely, we observed no multiplication of CPR following the passage made on the 6th day of the initial enrichment (Fig. 2). This test confirms the viability of *Saccharibacteria* cells attached to S. odontolytica bacteria and the success of CPR coculture using this protocol.

However, coculturing of the pellet of a third sample (starting *C_T_* = 18.92) with the three *Streptomyces* strains did not render similar results. The *C_T_* values remained stable afterwards for 8 days. Even the two passages did not increase the *C_T_* values under aerobic and anaerobic conditions. Thus, the *Saccharibacteria* cells did not multiply following their association with this new bacterial host (Fig. 2).

Finally, after 48 h of coculture, 50 to 100 μl of each enrichment broth was deposited on Columbia agar supplemented with 5% sheep blood (COS), SHI agar (6), and brain heart infusion (BHI) agar supplemented with 10% sheep blood. Each anaerobically isolated colony was tested by qPCR. Our qPCR system could not identify positive colonies. For greater precision, a standard PCR test was performed, and all colonies were negative for *Saccharibacteria*. The matrix-assisted laser desorption ionization–time of flight mass spectrometry (MALDI-TOF MS) test identified most of the isolated colonies as S. odontolytica (formerly Actinomyces odontolyticus)/Streptococcus oralis with a high score (>1.9). This score indicates the absence of foreign proteins (such as *Saccharibacteria* proteins) in each colony that can affect the spectra related to each known bacterium.

### *Saccharibacteria* cell imaging by electron microscopy.

Each initial sample was observed using electron microscopy (Hitachi TM4000Plus and SU5000 microscopes). We noticed a strong presence of biofilm and many coccus microbes attached to the external surface of several bacterial forms (bacilli and cocci). The sizes of these particles ranged from 100 to 400 nm, which corresponds to the described size of CPR members (Fig. 3).

However, following the filtration/centrifugation of the initial enrichment step, we were able to observe single and detached coccus forms, with no association with any bacterial host (Fig. 3). The sizes of these particles were similar to those observed in the original samples and much smaller than the known coccus bacteria (Staphylococcus spp. and Streptococcus spp., for example). These observations, along with the molecular results, confirm that *Saccharibacteria* cells were well separated from their bacterial hosts (Fig. 3).

Finally, as a negative control, a microscope slide for each host strain used (the host strains were negative in our *Saccharibacteria* qPCR system) was viewed in every step using the two electron microscopes; we were unable to detect any form with a size similar to that of CPR cells. However, round-shaped cells (1 to 2 per bacterial cell) appeared on the surface of these strains on the second day of their coculture with the enriched/filtered *Saccharibacteria* spp. (Fig. 3), and single *Saccharibacteria* and S. odontolytica cells continued to be observed. Hence, a physical association between *Saccharibacteria* and its host appeared. There were, therefore, bacteria that did not harbor CPR and other bacteria that were carriers of a maximum of one or two *Saccharibacteria* cells. The observations on day 4 and day 6 showed the same results.

### Genomic sequencing and description.

**FIG 4:**
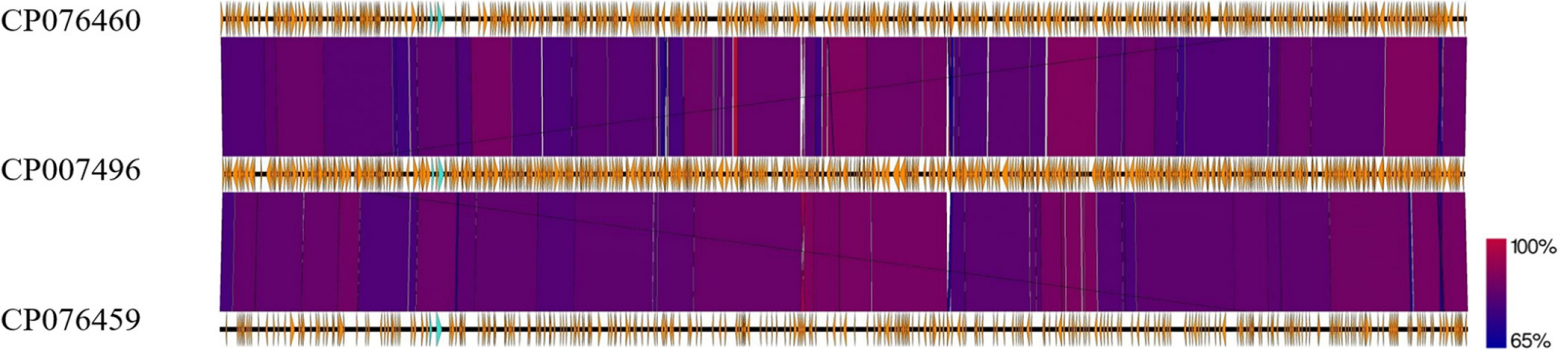
Graphic representation showing the genomic comparison between “*Candidatus* Minimicrobia naudis” (CP076460), “*Candidatus* Nanosynbacter lyticus” (TM7x) (reference genome CP007496) and “*Candidatus* Minimicrobia vallesae” (CP076459). This representation was generated using the Easyfig version 2.2.5 online tool.

**FIG 5:**
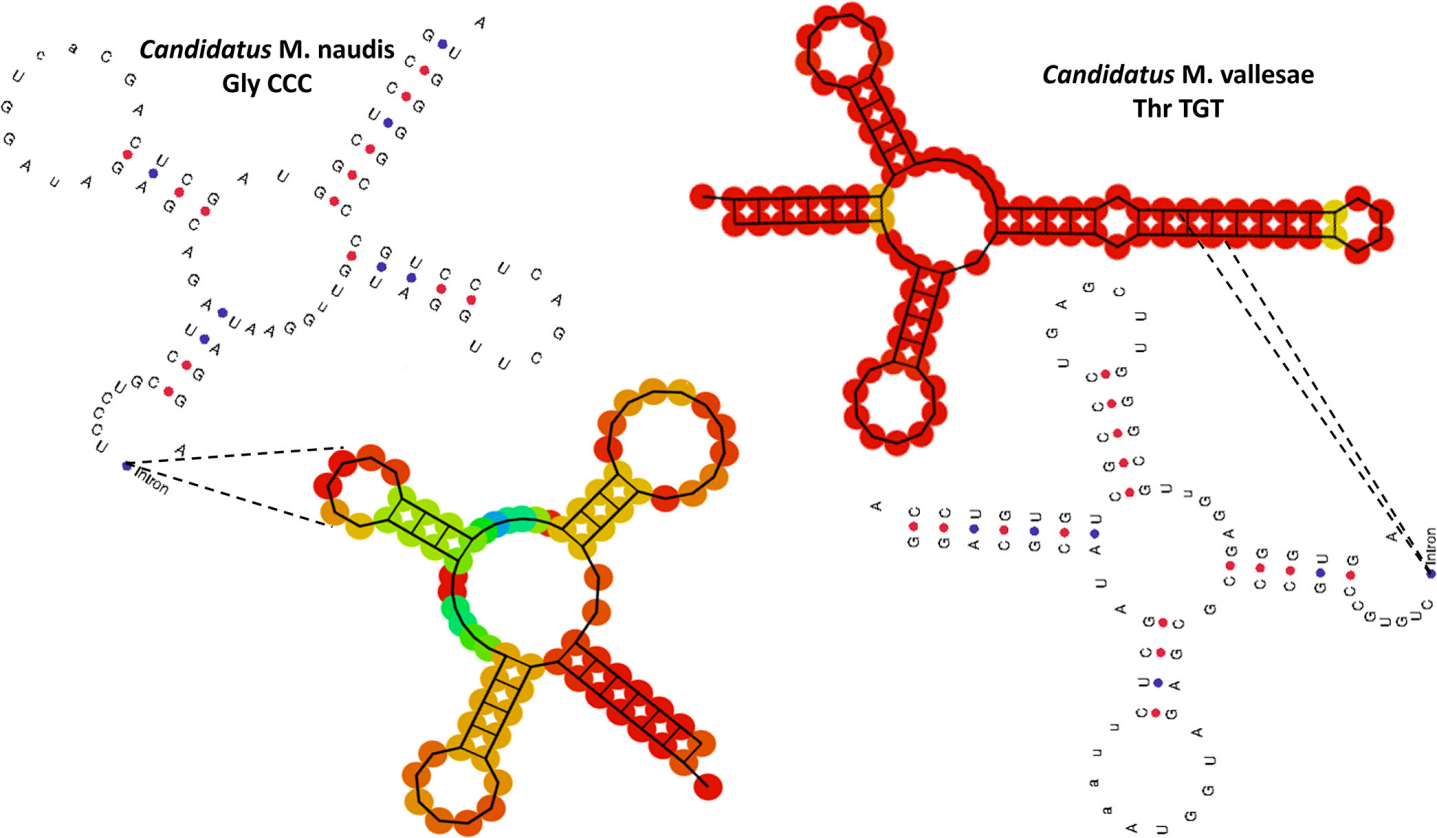
Two-dimensional representation of tRNAs with intronic sequences detected in “*Candidatus* Minimicrobia naudis” and “*Candidatus* Minimicrobia vallesae.”

For each DNA sample, the total Illumina and Nanopore reads were mapped against the *Saccharibacteria* reference genome (TM7x genome) using the CLC genomics 7 server. The filtration protocol, combined with the pretreatment extraction, allowed us to cover the entire TM7x genome (100%) in each DNA sample. Using long-range PCR, we obtained two complete genomes representing two new *Saccharibacteria* species. The first genome (named “*Candidatus* Minimicrobia naudis”) had a length of 708,351 bp with 43.9% G+C content. It had 1,324 protein coding genes that included 792 hypothetical proteins (59.81%). Similarly, the second sequenced genome (“*Candidatus* Minimicrobia vallesae”) had a length of 706,973 bp and 43.7% G+C content, and 48.97% of its protein coding genes (*n* = 1,017) corresponded to hypothetical proteins (Table S1 in the supplemental material). In addition, according to the proteomic analysis, 719 and 618 protein-coding genes of “*Ca*. Minimicrobia naudis” and “*Ca*. Minimicrobia vallesae,” respectively, were assigned to Clusters of Orthologous Groups (COG) categories (Fig. S1 and Table S2). We did not detect any proteins belonging to the following COG categories: B, Q, W, X, Y, and Z. A graphic circular map for each genome is presented in Fig. S2. A genomic comparison between our two genomes and that of TM7x (as the reference genome) using Easyfig version 2.2.5 is presented in Figure 4. In addition, recent studies have shown the presence of introns in the tRNA of CPR members (12). Here, we identified one tRNA per genome that contained an intronic sequence, Gly CCC for “*Ca*. Minimicrobia naudis” and Thr TGT for “*Ca*. Minimicrobia vallesae” (Fig. 5). We did not find any nonribosomal peptide synthetase/polyketide synthase (NRPS/PKS) clusters or insertion sequences in either genome. In addition, according to Maatouk et al., we applied the same adapted strategy to predict the antibiotic resistance genes in these genomes (15). “*Ca*. Minimicrobia naudis” was resistant to glycopeptide and tetracycline. Likewise, we found resistance genes for glycopeptide, tetracycline, and macrolides-lincosamides-streptogramin in the “*Ca*. Minimicrobia vallesae” genome (15). Finally, for the pilus secretion systems, we found type II, IV, and VI pilus secretion systems in both genomes and type I in “*Ca*. Minimicrobia vallesae” only.

**FIG 6:**
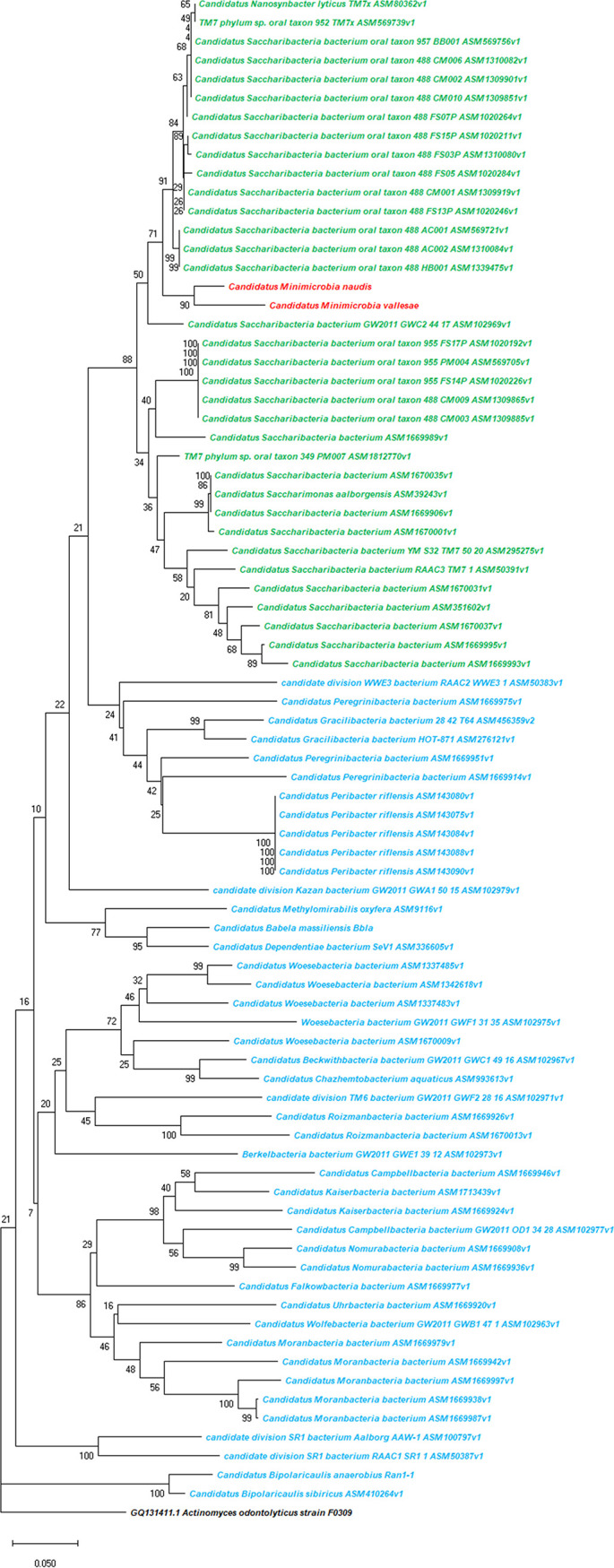
Unrooted phylogenetic tree shows the analysis of the 16S rRNA genes of all available *Saccharibacteria* complete genomes (marked in green), “*Candidatus* Minimicrobia naudis” (marked in red), “*Candidatus* Minimicrobia vallesae” (marked in red), and all available non-*Saccharibacteria* CPR complete genomes (marked in blue). The 16S rRNA gene of Schaalia odontolytica was used as the outgroup. This tree was generated using MegaX.

**FIG 7:**
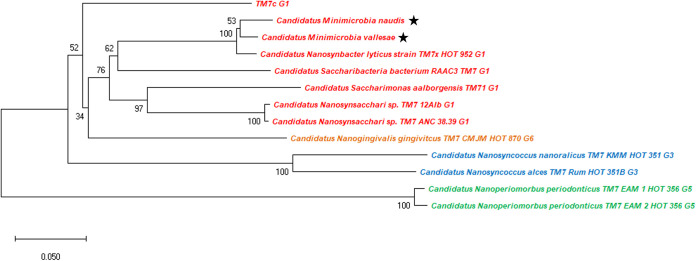
Unrooted phylogenetic tree based on concatenated ribosomal proteins of “*Candidatus* Minimicrobia naudis,” “*Candidatus* Minimicrobia vallesae,” and CPR species belong to clades G1, G3, G5, and G6. Species belonging to clade G1 are marked in red, to G3 in blue, to G5 in green, and to G6 in orange. Stars indicate genomes sequenced in this study.

For taxogenomic classification, the phylogenetic tree based on 16S rRNA and the whole-genome sequence analyses show that our two new *Minimicrobia* species belong to the superphylum *Saccharibacteria* (Fig. 6). In addition, the analyses of 16S rRNA described above and the phylogenetic tree based on concatenated ribosomal proteins according to McLean et al. show that our two new species belong to clade G1 of *Saccharibacteria* oral species (Fig. 7) (30, 31). The maximum orthologous average nucleotide identity (OrthoANI) values were 84.2412% for “*Ca*. Minimicrobia naudis” with TM7 phylum sp. oral taxon 952 (ASM569739v1), 84.0275% for “*Ca*. Minimicrobia vallesae” with “*Ca*. Nanosynbacter lyticus” (ASM80362v1), and 90.7707% between them (Fig. S3 and Table S3). Moreover, the maximum average amino acid identities (AAIs) were 89.6% and 93.9%, respectively, for “*Ca*. Minimicrobia naudis” and “*Ca*. Minimicrobia vallesae” with TM7 phylum sp. oral taxon 352 (ASM784539v1). Likewise, digital DNA-DNA hybridization showed that our described genomes had the highest values (26.01% for “*Ca*. Minimicrobia naudis” and 25.3% for “*Ca*. Minimicrobia vallesae”) with “*Candidatus* Saccharibacteria” bacterium oral taxon 955 (ASM1020192v1) and TM7 phylum sp. oral taxon 952 (ASM569739v1), respectively. The percentage between them was 41.7% (39.2 to 44.2 confidence interval). According to these values, and using the threshold proposed by Stackbrandt and Ebers to define a new bacterial species and as proposed by McLean et al. (31), we defined “*Candidatus* Minimicrobia naudis” and “*Candidatus* Minimicrobia vallesae” as two new CPR species belonging to superphylum: Saccharibacteria, Class: Saccharimonia, Order: Nanosynbacterales, Family: Nanosynbacteraceae, and to a new genus named: Minimicrobia.

Etymology: Minimicrobia (Mini – Microbe), a small microbe (Latin noun), naudis and vallesae (Latin nouns): in honor of Mrs. Naud and Mrs. Valles, French microbiologists.

**FIG 8:**
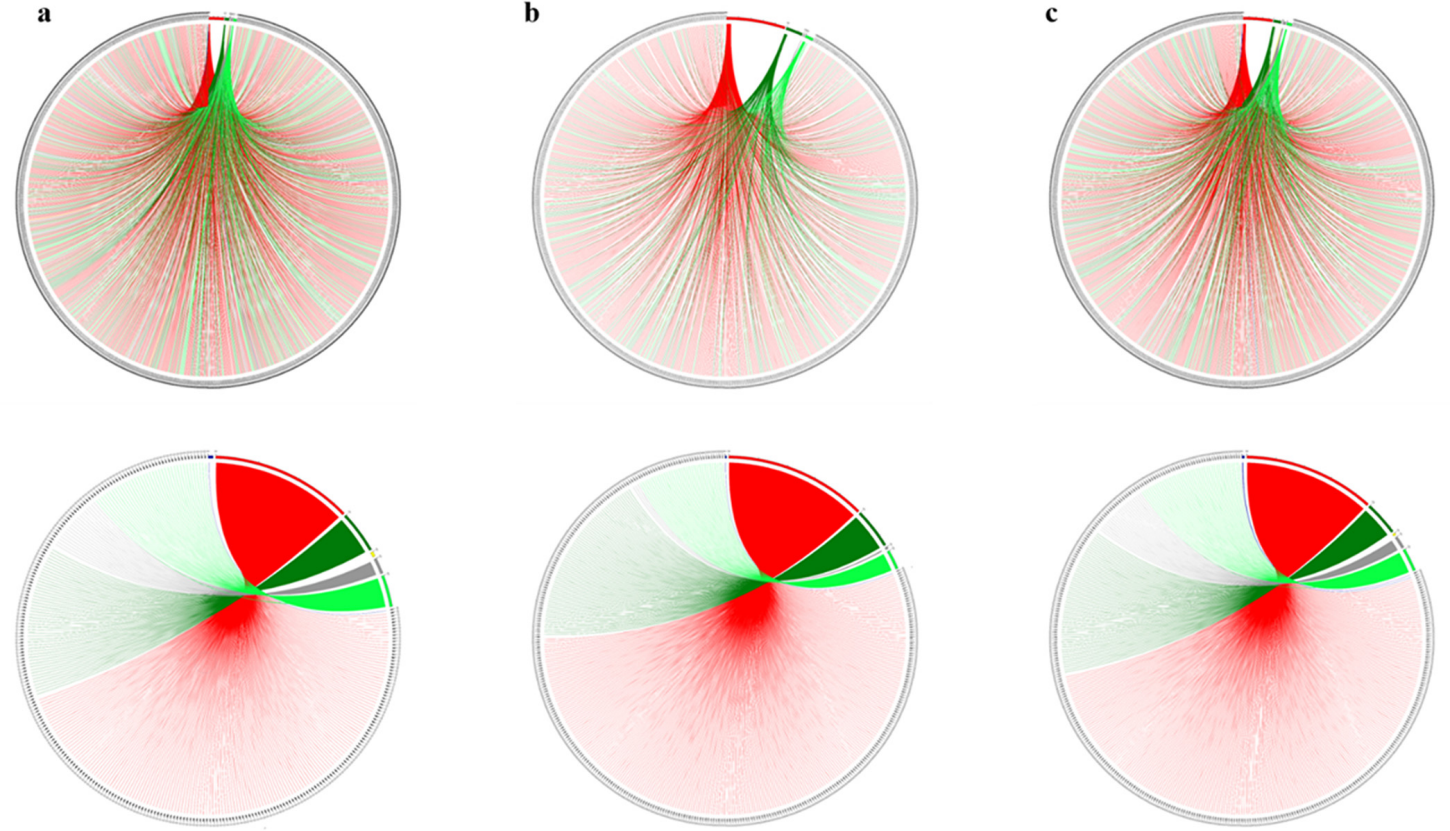
Rhizomal illustration presenting the mosaicism of each genome used. (a) “*Candidatus* Minimicrobia naudis.” (b) “*Candidatus* Nanosynbacter lyticus” (TM7x, reference genome). (c) “*Candidatus* Minimicrobia vallesae.” Each protein-encoding gene is represented by a curve, colored according to its origin as follows: bacterial origin in red, CPR non-*Saccharibacteria* phylum origin in dark green, *Saccharibacteria* phylum origin in light green, eukaryotic origin in yellow, archaeal origin in dark blue, and ORFans in gray. In the top row, each curve represents a protein-encoding gene, arranged in the figure by order. For the bottom row, protein-encoding genes belonging to the same origin are arranged together. Figures were constructed using the Circos tool.

According to the taxonomic affiliation of each *Saccharibacteria* sequence, the origin of each coding gene was determined. The evolutionary history of each genome is presented here based on all genomic sequences belonging to the repertoire of coding genes. We obtained a particular mosaicism for both “*Ca*. Minimicrobia naudis” and “*Ca*. Minimicrobia vallesae” (4), similar to one another and comparable to that of the reference genome (Fig. 8) (4).

For each genome, we found a prevalence of sequences of bacterial and CPR origins (45.6% and 42.6%, respectively, for “*Ca*. Minimicrobia naudis” and 48.4% and 45.4% for “*Ca*. Minimicrobia vallesae,” respectively). Among the sequences of CPR origin, a large percentage is unique to the superphylum *Saccharibacteria* (an average of 31% in each genome). However, we also detected some eukaryotic and archaean sequences in each genome (0.32% and 0.24%, respectively, for “*Ca*. Minimicrobia naudis” and 0.16% and 0.3%, respectively, for “*Ca*. Minimicrobia vallesae”) (Fig. 8) (4).

## DISCUSSION

The oral microbiota is known as the most complex human microbiota. It has been estimated that it may contain more than 775 microbial species (25). In addition, following the initial inclusion of CPR in the tree of life, different metagenomics studies have shown that the *Saccharibacteria* superphylum is very abundant in humans and, more precisely, in the oral cavity (19). Therefore, this coculture protocol was mainly tested on sputum samples.

The quantification and viability of *Saccharibacteria* cells have been tested by standard PCR in a number of studies (1, 6, 28, 32). Different sets of primers targeting the 16S rRNA have been identified as universal for this superphylum (32). According to these results, *Saccharibacteria* microbes were considered viable if the PCR was still positive after five passages (1). This method increases the risk of false-positive results due to amplifying DNA from dead microorganisms and/or misquantification. Here, we developed a TaqMan real-time qPCR system that was, for the first time, specific for the *Saccharibacteria* superphylum, enabling us to detect and quantify these microbes in any sample of interest. Compared to other published qPCR systems (29, 32), our system is the only one able to quantify members of the *Saccharibacteria* superphylum using a nucleotide probe. This probe hybridizes specifically to a conserved region of the 23S rRNA gene, which is in general more specific than the systems based on SYBR green as fluorescence (33). In addition, after the *in silico* analyses, 97.12% of all available *Saccharibacteria* (human and environmental sources) sequences had the same conserved amplified region and could be amplified by our system. Moreover, it is important to note that the only unamplified genome is from an environmental origin. So, until this date, this system is 100% specific for all *Saccharibacteria* spp. recovered from a human microbiome.

Because the DNA quantification (partial quantification) by this system is based only on *C_T_* values, it only allows us to compare samples/culture trials and to determine which ones are more concentrated than others (i.e., those with the lowest *C_T_* values). As we do not have a pure culture (pure *Saccharibacteria* colonies on agar), it is difficult to determine an exact starting concentration (copy/ml versus McFarland), as is usually done for bacteria and fungi (33).

Following our results, very low *C_T_* values were obtained from fresh sputa, confirming their abundance in the oral microbiota (19). Second, *Saccharibacteria* members have not yet been cultivated in pure culture. Their identification on agar media or by MALDI-TOF MS is currently impossible. The use of this system, followed by metagenomics analysis, therefore enables this superphylum to be screened in any sample and, in the future, may lead to greater precision regarding their prevalence in humans and in environmental samples.

In this study, in line with several others (1, 2, 6), we confirmed that *Saccharibacteria* cells (CPR cells in general) can detach themselves from their natural host bacteria following continuous agitation. They can then adapt to another host to multiply (1). Following a coculture of S. odontolytica strains with filtered *Saccharibacteria* cells, the *C_T_* values decreased after 2 days, which explains their persistence and viability in liquid medium. However, the *C_T_* values remained stable between days 2 and 8. It could, therefore, be suggested that the nutrients needed by CPR cells had already been consumed and/or the metabolic and nutrient transport between the host and the guest had entered a standby stage, and hence, we were unable to detect further multiplication.

Nutritional supplementation of this complex (renewal-of-enrichment passage at day 2) restored these activities. Two criteria should therefore be considered to keep CPR at the multiplying stage: having a suitable host and a well-renewed enriched medium. In addition, and as suggested by He et al., CPR accompanies *Schaalia* spp. in stable long-term infections due to the adaptation and rapid evolution of its host (6). Moreover, it is thought that, on day 6 of culture, the CPR were dead, and only the DNA of the dead cells was amplified. Therefore, we failed to decrease the *C_T_* value after a passage from the sixth day of initial culture. The protocol optimized in this study therefore guarantees high protection and easy enrichment/filtration of the CPR and ensures very sensitive monitoring of their viability by electron microscopy and qPCR. It also provides the *Saccharibacteria* with an enriched nutrient complex, especially with the addition of pig gastric mucin (2) during the host infection stage. This protocol could be used to search for other bacterial hosts not yet described for CPR.

It is known that the physical sizes of members of the CPR are between 100 and 300 nm, so we limited the filtration here to 0.45 μm, to avoid losing a quantity of CPR between 0.22 μm and 0.3 μm. Therefore, our metagenomics analyses of the filtrate showed some contaminations of sequences belonging to Streptococcus and *Veillonella* species that may pass through the filters (either DNAs or cells) (34). However, most of the reads still correspond to the superphylum *Saccharibacteria*/TM7 (≈84%).

Furthermore, we were unable to isolate a positive colony, as demonstrated by our real-time PCR system. In following a deposit of the starting sample and the filtrate mixed with *Schaalia* spp., all colonies were negative in real-time PCR and electron microscopy. A recent study showed that the use of reverse genomics methods was successful in producing *Saccharibacteria*-positive colonies (28). This method is based on a target antibody that only picks up *Saccharibacteria* with their hosts (28). In our assay, other microorganisms were able to pass through the 0.45-μm filter. We suggest that the requirements of *Saccharibacteria* members, their fragility and/or the presence of other microorganisms in the filtrate (Streptococcus oralis for example) prevented their multiplication on a solid medium, even though several enriched media were tried (COS and supplemented BHI and SHI agars). It would, therefore, be interesting to find universal epitopes common to all known *Saccharibacteria*, rather than based on one or two genomes, to facilitate culturing them on solid media and sorting them using flow cytometry.

It is known that *Saccharibacteria* members interact with S. odontolytica to multiply in an exosymbiotic (or exoparasitic) relationship, in stable long-term infections between these two microorganisms. Furthermore, different studies have suggested that *Saccharibacteria* spp. can adapt to other bacteria, such as *Arachnia* spp. for example (1). Here, the infection of *Streptomyces* spp. by enriched *Saccharibacteria* cells was not successful in terms of their multiplication, indicating that the association between these microorganisms is not appropriate to a nutrient transfer from the host bacterium to the CPR cells. Hence, *Streptomyces* cannot be considered one of the hosts of the identified *Saccharibacteria* species. Finally, this protocol extends the described diversity of CPR to date. It enabled us to recover two new species belonging to the superphylum *Saccharibacteria*. Both species are unique, and they are similar in size to those described in the literature but have very divergent sequences (the maximum OrthoANI/AAI and DDH values are very low). In addition, we found a tRNA with intronic sequences in each genome, which has recently been described in CPR genomes (12).

Concerning their origin, the presence of archaeal/eukaryotic sequences suggests the presence of an interaction between these microorganisms in their shared niche (4, 35, 36). The mosaic structure of CPR in general gives them a unique characteristic, comparable to one another and different from other microbial domains (4).

In conclusion, our developed protocol allowed us to coculture and sequence new *Saccharibacteria* species and to maintain their viability as demonstrated by molecular quantification and electron microscopic imaging. Moreover, the use of our TaqMan probe as a fluorescence *in situ* hybridization probe could be interesting in future studies. This step could give more visual evidence for the presence of CPR and their interactions with their hosts, along with images from electron microscopy. In addition, it is important to test this protocol on different clinical samples (vaginal, fecal, urinary, etc.) in the context of improving our knowledge of the physiology and physiopathology of this CPR superphylum.

## MATERIALS AND METHODS

### Sample collection and ethics statement.

Twenty-eight sputum samples were collected at La Timone University Hospital (Assistance Publique-Hôpitaux Marseille [AP-HM]) from routine laboratory diagnostics. Research analyses were only performed on surplus samples, once laboratory diagnostic procedures had been initiated. The patients were informed that their samples may be used for research purposes and retained the right to oppose this use. Given that this study did not involve specific collection of samples or use medical/personal data from patients, and according to French law (the Jardé’s law), neither institutional ethical approval nor individual patient consent was required for this noninvasive study (Loi no 2012–300 du 5 mars 2012 and Décret no 2016–1537 du 16 novembre 2016 published in the *Journal Officiel de la République Française*).

Concerning the three samples used for our adapted protocol, each 2 ml was diluted in 1 ml of transport medium (composed of 0.1 g MgCl_2_, 0.2 g KH_2_PO_4_, 1.15 g NaCl, 1 g Na_2_HP_4_, 1 g ascorbic acid, 1 g uric acid, and 1 g glutathione per 1 liter of deionized water, pH 7.5). All tested samples were stored under anaerobic conditions.

### Isolation of *Saccharibacteria* spp. and culture conditions.

In a hemoculture tube, we diluted 1 ml of each sputum sample in 39 ml of enriched broth (37 g BHI, 10 g yeast extract, 10 mg hemin, and 50 μl vitamin K per 1,000 ml tryptic soy broth, final pH = 7; bioMérieux, Marcy-l’Etoile, France) at 37°C and in an atmosphere of 85% N_2_, 10% CO_2_, and 5% H_2._ Each culture was performed in an anaerobic chamber (Coy) for 7 days with agitation (300 rpm) to separate the *Saccharibacteria* cells present from their bacterial hosts. After 7 days of enrichment and agitation, the broth was filtered at 0.8 μm and 0.45 μm, respectively, to eliminate large particles and associated cultured bacteria. For greater cell concentration, an ultracentrifugation of 100,000 × *g* was then performed for 2 h at 4°C. The pellet (which was sometimes invisible) was resuspended in 2.5 ml of the enrichment broth mentioned above, supplemented with 2.5 g/liter of pig gastric mucin (2).

In addition, we prepared 1-McFarland solutions of six Schaalia odontolytica strains (previously known as Actinomyces odontolyticus), isolated from a human oral cavity, in physiological water. For each resuspended pellet, 200 μl was used for molecular biology analyses and the remaining quantity was cultured with 0.1 ml of the *Schaalia odontolytica* strain solution for 7 days in a Hungate tube with no agitation under the same anaerobic conditions described above. After 48 h of culture, 50 to 100 μl of each enrichment broth was deposited on COS agar, SHI agar, and BHI agar (bioMérieux, Marcy-l’Etoile, France) supplemented by 10% sheep blood and 2.5 pig gastric mucin, each under anaerobic conditions (Fig. 1). The same culture protocol described above was also tested on other samples by mixing the filtrate with 1 McFarland of three *Streptomyces* species strains (Streptomyces cattleya strain DSM 46488, Streptomyces massiliensis, and Streptomyces rochei) isolated from the human gut, separately, under aerobic and anaerobic conditions (Fig. 1).

### *Saccharibacteria* quantification.

To evaluate *Saccharibacteria* coculture, we designated a real-time qPCR system for quantification. To do so, we selected all of the *Saccharibacteria* complete genomes available in NCBI on 1 December 2020 (*n* = 25). Based on the conserved ribosomal genes, a multiple alignment of the 23S rRNA genes was performed to determine the conserved zones. We consequently selected SacchariF (GGCTTATAGCGCCCAATAG) as a forward primer, SacchariR (CGGATATAAACCGAACTGTC) as a reverse primer, and SacchariP (6-FAM [6-carboxyfluorescein]-CATAGACGGCGCTGTTTGGCAC-TAMRA [6-carboxytetramethylrhodamine]) as a TaqMan probe.

The specificity of this system was confirmed *in silico* by BLASTn against the nr database and *in vitro* against 50 bacterial species, 70 *Candida* strains (33), and 25 stool samples that had previously tested negative with the specific *Saccharibacteria* standard PCR (580-F/1177-R) (37). Finally, our qPCR was tested *in silico* against 10 additional *Saccharibacteria* complete genomes that became available on NCBI between 1 December 2020 and 1 June 2021.

To improve the extraction of *Saccharibacteria* DNA, several pretreatments were performed for each tested sample and culture condition. For deglycosylation, each 180-μl sample/*Saccharibacteria* coculture was treated with the Endo Hf kit (catalog number P0703L; New England Biolabs, Evry, France), as follows. Three microliters of each reagent was added to the sample, and the sample was incubated for 1 h at room temperature and then 1 h at 37°C. We then added 10 μl lysozyme for 2 h and 10 μl proteinase K for 12 h at 56°C, which was followed by a 1-min disruption with glass powder using Fast-Prep. We used the EZ1 biorobot (Qiagen, Tokyo, Japan) for the automated extraction, using the EZ1 DNA tissue kit (Qiagen, Hilden, Germany) and the bacterial protocol card. After extraction, each extracted DNA was eluted in 50 μl EZ1 elution buffer. A PCR quantification test (qPCR) was then performed in duplicate on each sample before culture and every 48 h after infecting Schaalia odontolytica strains/*Streptomyces* species strains with enriched/filtered *Saccharibacteria* cells. For this purpose, we used the CFX96 connect real-time PCR detection system (BIO-RAD, Life Science, Marnes-la-Coquette, France) using TaqMan technology (Fig. 1). The qPCRs were carried out according to the following protocol: 2 min of incubation at 50°C, 15 min of activation at 95°C, followed by 40 cycles of 5 s at 95°C and 30 s at 60°C for DNA amplification, and then a final step at 45°C for 30 s. We prepared each qPCR mixture in a 20-μl total volume containing 10 μl of QuantiTect assay primers, 2 μl of sterile water, 1 μl of each primer, 1 μl of probe, and 5 μl of each DNA (33). In addition, to confirm the specificity of the qPCR, each amplicon was sequenced using the Sanger method and analyzed by BLASTn against the nr database.

### Bacterial and CPR imaging.

All specimens or samples were fixed in 2.5% glutaraldehyde solution and were deposited by cytocentrifugation on cytospin slides, followed by staining with a 1% phosphotungstic acid aqueous solution (pH = 7) for 3 min. All samples were then sputter coated with a 10-nm-thick layer of platinum to reduce charging of the imaged samples.

For image acquisition, we first used Hitachi’s TM4000Plus tabletop SEM, approximately 60 cm in height and 33 cm wide, to evaluate bacterial structure. We used backscattered-electron imaging for detection. The voltage of acceleration was 10 kV, and magnifications varied from ×250 to ×7,000. Using the same accelerating voltage, we then used Hitachi’s SU5000 SEM for the images with higher resolution and magnifications. Magnifications varied from ×5,000 to ×15,000. The evacuation time after loading specimens into the SEM chamber was less than 2 min. All cocultures of samples/*Saccharibacteria* cells were acquired using the same acquisition settings regarding magnification, intensity, and voltage mode. Here, each microbial form that presented a coccus shape and a physical size between 100 and 400 nm and was outside or attached to a bacterium was considered a CPR cell.

### Next-generation sequencing.

Extracted DNA was sequenced using two different methods, with the first on the MiSeq (Illumina, Inc., San Diego, CA, USA) using the Nextera XT DNA sample prep kit (Illumina) with the paired-end strategy. The tagmentation step fragmented and tagged each extracted DNA to prepare the paired-end library. A limited PCR amplification (12 cycles) was then performed to complete the tag adapters and to introduce dual-index barcodes. DNA was then purified on AMPure XP beads (Beckman Coulter, Inc., Fullerton, CA, USA). In addition, according to the Nextera XT protocol (Illumina), all libraries were normalized on specific beads. We then pooled all libraries into one library for DNA sequencing on the MiSeq. The pooled single-strand library was loaded onto the reagent cartridge and then onto the instrument along with the flow cell. Automated cluster generation and paired-end sequencing with dual index reads were performed in a single 39-h run in 2 × 250 bp.

The Oxford Nanopore method was then performed for 1D genomic DNA sequencing on the GridION device, using the SQK-LSK109 kit. A library was constructed from 1 μg genomic DNA without fragmentation and end repair. Adapters were ligated to both ends of the genomic DNA. After purification on AMPure XP beads (Beckman Coulter, Inc., Fullerton, CA, USA), the library was quantified by a Qubit assay with the high-sensitivity kit (Life Technologies, Carlsbad, CA, USA). We detected active pores for sequencing, and the WIMP workflow was chosen for live bioinformatic analyses.

### Genomic description.

For each sample/filtered *Saccharibacteria* DNA sequence, the quality of each Illumina and Oxford Nanopore read was checked by FastQC and trimmed using Trimmomatic version 0.36.6. We merged all the reads that corresponded to a given sample (this protocol was applied to one sputum sample and then confirmed on a second). Each group of reads was mapped against the reference *Saccharibacteria* genome (“*Ca*. Nanosynbacter lyticus” genome, available in NCBI under accession number ASM80362v1) using CLC Genomics Workbench version 7. We used the default parameters except for the length fraction (reduced to 0.3) and the similarity fraction (reduced to 0.5). Mapped reads were assembled using SPAdes software, version 3.13.0 (38), with the default options. For this step, we only kept contigs with a minimum size of 400 bp. Each contig was then analyzed by BLASTn against the nr database, and we only kept contigs which matched sequences corresponding to *Saccharibacteria* spp. All selected fasta sequences were then mapped against the TM7x (“*Ca*. Nanosynbacter lyticus”) genome (accession number ASM80362v1), using the same criteria mentioned above to generate the sequenced *Saccharibacteria* genome with no contamination by any bacterial/eukaryotic sequences.

To complete our sequenced genomes (to fill in the gaps), we designed primers around each gap to perform long-range PCR. Each PCR product (amplicon) was then sequenced using the Oxford Nanopore method and mapped against the contig to link them. These genomes were deposited in GenBank as complete genomes under accession numbers CP076459 and CP076460. Then, coding and noncoding genes, hypothetical proteins, coding sequences, and rRNA were predicted using Prokka (39). tRNA genes were predicted by tRNA SCAN SE, using the default option and all available sequence sources (40). Proteomes were predicted with BLASTp (E value of 0.001, minimum coverage and identity of 70% and 30%, respectively) against the Cluster of Orthologous Groups database (41). Antibiotic resistance genes were then predicted using the adapted strategy for CPR that was recently described in Maatouk et al. (15). Similarly, we looked for the presence of NRPS-PKS using BLASTp against the Non-Ribosomal Peptide and Polyketide Synthase PURified (NRPPUR) database (42) In addition, in order to detect lateral sequence transfers between our species and their host (presence of transposon/integron), a screening for IS sequences was performed by BLASTn and BLASTp against the ISfinder online tool (43). *Saccharibacteria* members are known to have protein secretion systems (pili) that attach to the external membrane of their host. For this purpose, we screened our assembled genomes against the MacSyDB/TXSSdb online database (44) to detect all protein secretion systems that were presented. An additional genomic comparison between our genomes and the TM7x reference genome was then performed using Easyfig version 2.2.5 (45).

To determine the mosaicism and evolutionary history of each genome, we constructed a representative rhizome that showed the genetic exchange between our sequenced *Saccharibacteria* spp. and the other organisms (4). For this purpose, a BLASTp against the NCBI protein database was performed for each coding gene. Any protein that did not match with any sequence was considered an ORFan (an open reading frame [ORF] with no detectable homology to other ORFs in a database). The remaining best hits were selected based on the following criteria: minimum identity and coverage of 20% and 30%, respectively, and maximum E value of 0.001, as previously described (4, 46). Rhizome representations were then constructed using the Circos software (47).

For taxonomic characterization, we selected for comparison all CPR (*Saccharibacteria* superphylum and others) complete genomes that were available in NCBI on 1 June 2020 (*n* = 81). A multiple alignment of 16S rRNA sequences was performed using MUSCLE software, and curated alignments were then used for the construction of a phylogenetic tree using the neighbor-joining method, with 500 bootstrap replicates, using the method of Felsenstein (30). In addition, we computed the evolutionary distance using the Jukes-Cantor method exactly as previously described (30). The tree was constructed using MEGA-X software. In addition, the degrees of genomic/proteomic similarity between our new species and all selected genomes were estimated using OrthoANI software and AAI-profiler, respectively. We also used the Genome-to-Genome Distance Calculator Web service to calculate the digital DNA-DNA hybridization (dDDH) values, with confidence intervals, according to recommended parameters, as previously described (48).

### Data availability.

The “*Candidatus* Minimicrobia vallesae” and “*Candidatus* Minimicrobia naudis” genomes were deposited in NCBI GenBank under accession numbers CP076459 and CP076460, respectively.
