# Supplementary material for: Adapted Protocol for Saccharibacteria Cocultivation: Two New Members Join the Club of Candidate Phyla Radiation
Source: Microbiol Spectr. 2021 Dec 22;9(3):e01069-21. doi: 10.1128/spectrum.01069-21 (PMC8694215; doi:10.1128/spectrum.01069-21)
Supplement: SUPPLEMENTAL FILE 1 — Supplemental material. Download SPECTRUM01069-21_Supp_1_seq10.pdf, PDF file, 3.3 MB [file spectrum01069-21_supp_1_seq10.pdf]

SUPPLEMENTAL MATERIAL

Figure S1: Graphic representation showing the distribution of functional classes of predicted genes according to the clusters of orthologous groups (COGs) of proteins of *Candidatus* Minimicrobia naudis and *Candidatus* Minimicrobia vallesae, among other *Saccharibacteria* complete genomes.

Figure S2: Graphic circular map of *Candidatus* Minimicrobia naudis and *Candidatus* Minimicrobia vallesae complete genomes. Maps were generating using the CG view online tool.

Figure S3: Heat map generated with OrthoANI values calculated using the OAT software between *Candidatus* Minimicrobia naudis and *Candidatus* Minimicrobia vallesae and all CPR complete genomes available on NCBI at 1 June 2021.

Table S1: Genomic characteristics of *Candidatus* Minimicrobia naudis and *Candidatus* Minimicrobia vallesae.

Table S2: Distribution of functional classes of predicted genes according to the clusters of orthologous groups (COGs) of proteins of *Candidatus* Minimicrobia naudis and *Candidatus* Minimicrobia vallesae, among other *Saccharibacteria* complete genomes.

Table S3: OrthoANI values calculated using OAT software between *Candidatus* Minimicrobia naudis and *Candidatus* Minimicrobia vallesae and all CPR complete genomes available on NCBI at 1 June 2021.

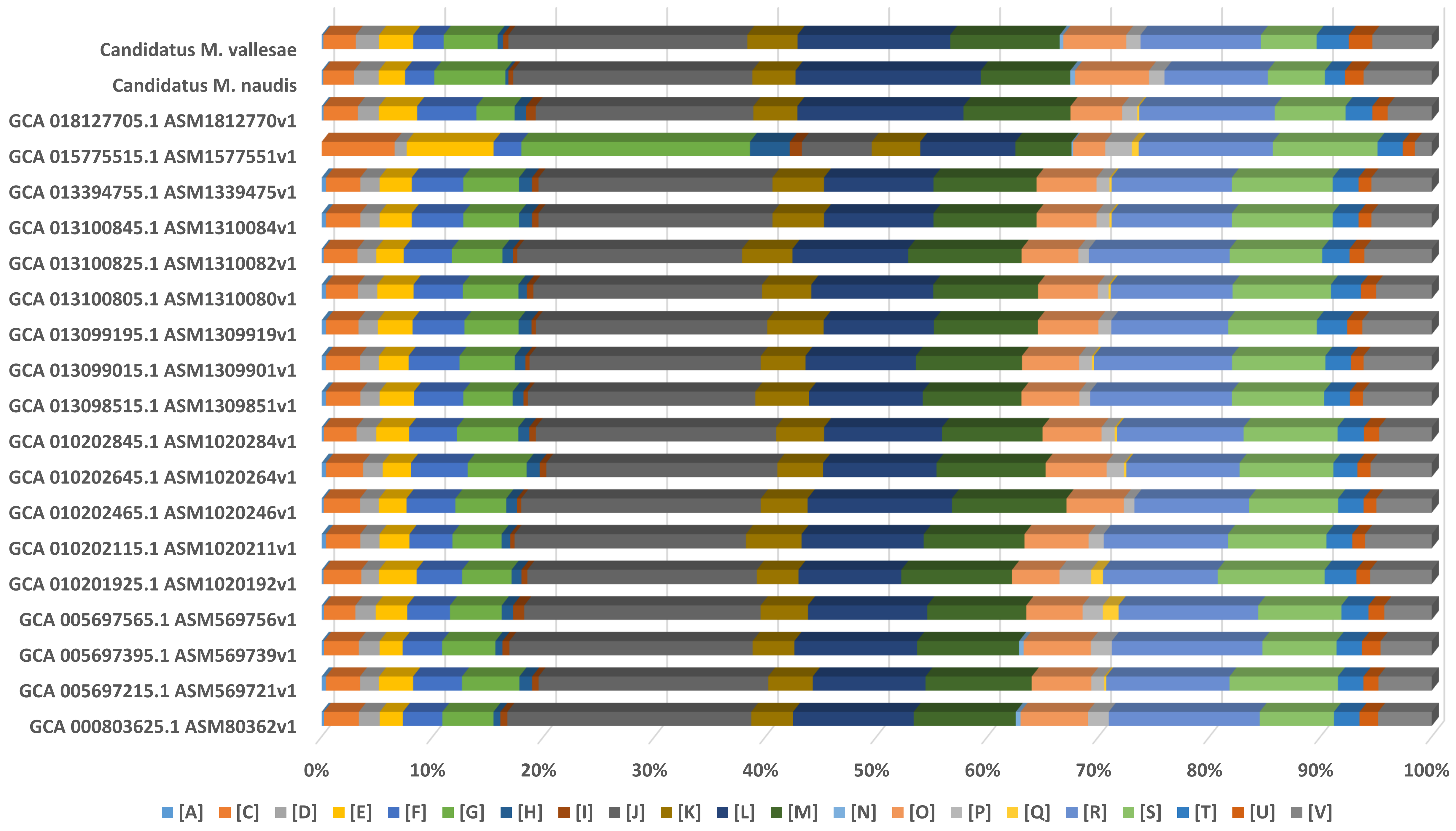

Candidatus M. vallesae
Candidatus M. naudis
GCA 018127705.1 ASM1812770v1
GCA 015775515.1 ASM1577551v1
GCA 013394755.1 ASM1339475v1
GCA 013100845.1 ASM1310084v1
GCA 013100825.1 ASM1310082v1
GCA 013100805.1 ASM1310080v1
GCA 013099195.1 ASM1309919v1
GCA 013099015.1 ASM1309901v1
GCA 013098515.1 ASM1309851v1
GCA 010202845.1 ASM1020284v1
GCA 010202645.1 ASM1020264v1
GCA 010202465.1 ASM1020246v1
GCA 010202115.1 ASM1020211v1
GCA 010201925.1 ASM1020192v1
GCA 005697565.1 ASM569756v1
GCA 005697395.1 ASM569739v1
GCA 005697215.1 ASM569721v1
GCA 000803625.1 ASM80362v1
0%
10%
20%
30%
40%
50%
60%
70%
80%
90%
100%
[A] [C] [D] [E] [F] [G] [H] [I] [J] [K] [L] [M] [N] [O] [P] [Q] [R] [S] [T] [U] [V]

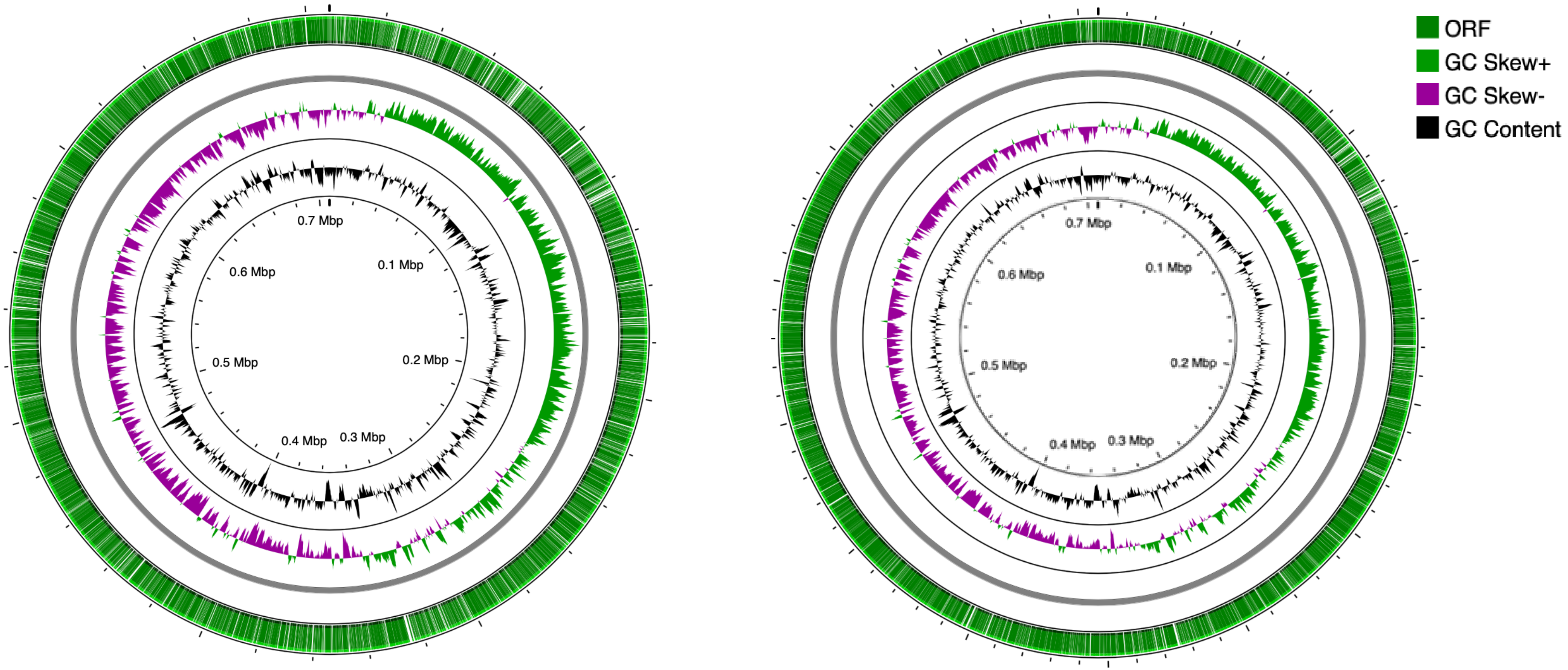

Candidatus Minimicrobia naudis complete genome

Candidatus Minimicrobia vallesae complete genome

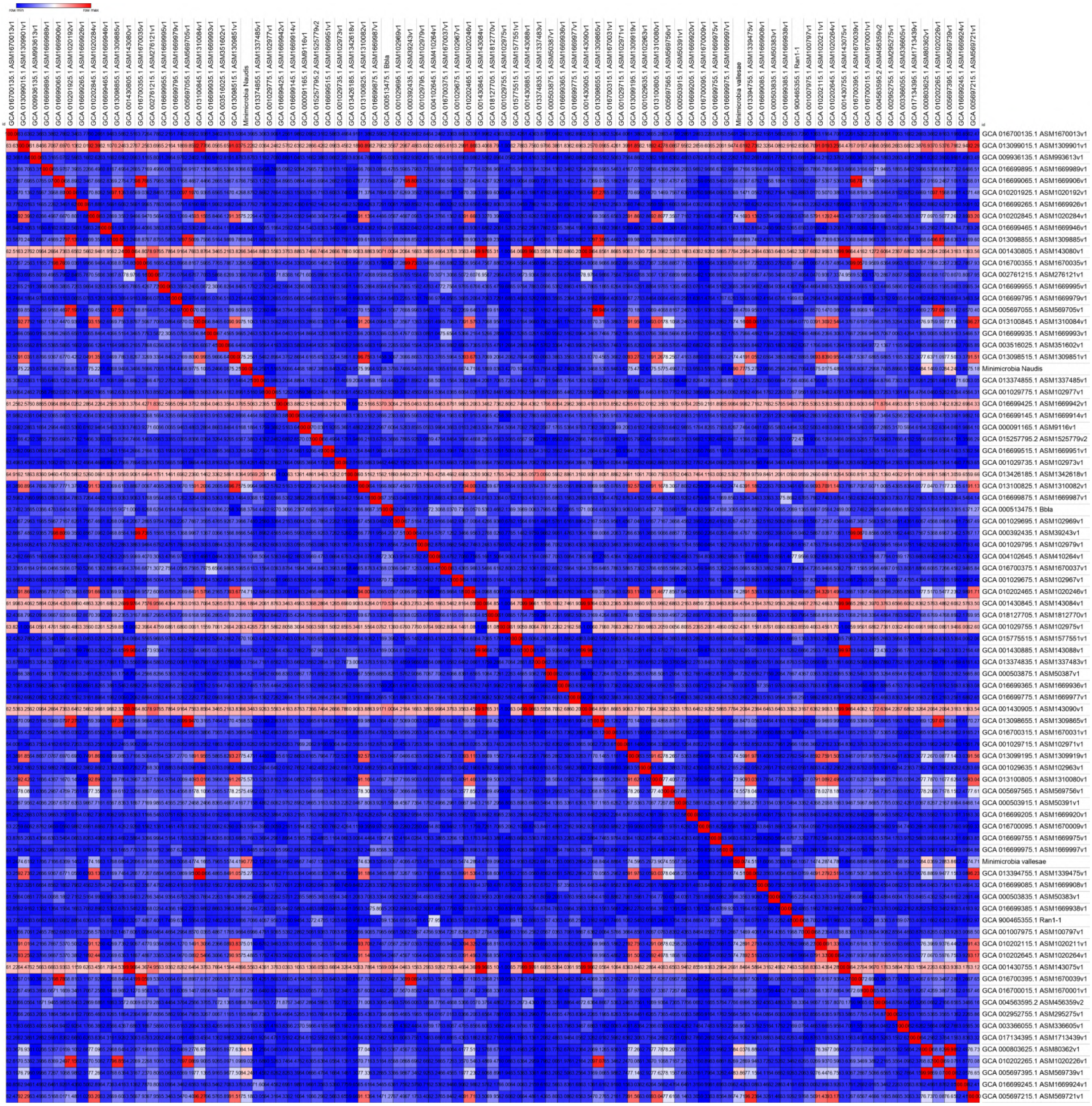
GCA 016700135.1 ASM1670013v1
GCA 013099015.1 ASM1309901v1
GCA 009936135.1 ASM993613v1
GCA 016699895.1 ASM1669989v1
GCA 016699065.1 ASM1669906v1
GCA 010201925.1 ASM1020192v1
GCA 016699265.1 ASM1669926v1
GCA 010202845.1 ASM1020284v1
GCA 016699465.1 ASM1669946v1
GCA 013098855.1 ASM1309885v1
GCA 001430805.1 ASM143080v1
GCA 016700355.1 ASM1670035v1
GCA 002761215.1 ASM276121v1
GCA 016699955.1 ASM1669995v1
GCA 016699795.1 ASM1669979v1
GCA 005697055.1 ASM569705v1
GCA 013100845.1 ASM1310084v1
GCA 016699935.1 ASM1669993v1
GCA 003516025.1 ASM351602v1
GCA 013098515.1 ASM1309851v1
Minimicrobia Naudis
GCA 013374855.1 ASM1337485v1
GCA 001029775.1 ASM102977v1
GCA 016699425.1 ASM1669942v1
GCA 016699145.1 ASM1669914v1
GCA 000091165.1 ASM9116v1
GCA 015257795.2 ASM1525779v2
GCA 016699515.1 ASM1669951v1
GCA 001029735.1 ASM102973v1
GCA 013426185.1 ASM1342618v1
GCA 013100825.1 ASM1310082v1
GCA 016699875.1 ASM1669987v1
GCA 000513475.1 Bbla
GCA 001029695.1 ASM102969v1
GCA 000392435.1 ASM39243v1
GCA 001029795.1 ASM102979v1
GCA 004102645.1 ASM410264v1
GCA 016700375.1 ASM1670037v1
GCA 001029675.1 ASM102967v1
GCA 010202465.1 ASM1020246v1
GCA 001430845.1 ASM143084v1
GCA 018127705.1 ASM1812770v1
GCA 001029755.1 ASM102975v1
GCA 015775515.1 ASM1577551v1
GCA 001430885.1 ASM143088v1
GCA 013374835.1 ASM1337483v1
GCA 000503875.1 ASM50387v1
GCA 016699365.1 ASM1669936v1
GCA 016699775.1 ASM1669977v1
GCA 001430905.1 ASM143090v1
GCA 013098655.1 ASM1309865v1
GCA 016700315.1 ASM1670031v1
GCA 001029715.1 ASM102971v1
GCA 013099195.1 ASM1309919v1
GCA 001029635.1 ASM102963v1
GCA 013100805.1 ASM1310080v1
GCA 005697565.1 ASM569756v1
GCA 000503915.1 ASM50391v1
GCA 016699205.1 ASM1669920v1
GCA 016700095.1 ASM1670009v1
GCA 016699755.1 ASM1669975v1
GCA 016699975.1 ASM1669997v1
Minimicrobia vallesae
GCA 013394755.1 ASM1339475v1
GCA 016699085.1 ASM1669908v1
GCA 000503835.1 ASM50383v1
GCA 016699385.1 ASM1669938v1
GCA 900465355.1 Ran1-1
GCA 001007975.1 ASM100797v1
GCA 010202115.1 ASM1020211v1
GCA 010202645.1 ASM1020264v1
GCA 001430755.1 ASM143075v1
GCA 016700395.1 ASM1670039v1
GCA 016700015.1 ASM1670001v1
GCA 004563595.2 ASM456359v2
GCA 002952755.1 ASM295275v1
GCA 003366055.1 ASM336605v1
GCA 017134395.1 ASM1713439v1
GCA 000803625.1 ASM80362v1
GCA 010202265.1 ASM1020226v1
GCA 005697395.1 ASM569739v1
GCA 016699245.1 ASM1669924v1
GCA 005697215.1 ASM569721v1
